# The Functional Role of the Periphery in Emotional Language Comprehension

**DOI:** 10.3389/fpsyg.2013.00294

**Published:** 2013-05-27

**Authors:** David A. Havas, James Matheson

**Affiliations:** ^1^Department of Psychology, University of Wisconsin-WhitewaterWhitewater, WI, USA; ^2^School of Cognitive Science, Hampshire CollegeAmherst, MA, USA

**Keywords:** embodied cognition, language comprehension, simulation, facial feedback, emotion, botox, motor control, constraint satisfaction

## Abstract

Language can impact emotion, even when it makes no reference to emotion states. For example, reading sentences with positive meanings (“The water park is refreshing on the hot summer day”) induces patterns of facial feedback congruent with the sentence emotionality (smiling), whereas sentences with negative meanings induce a frown. Moreover, blocking facial afference with botox selectively slows comprehension of emotional sentences. Therefore, theories of cognition should account for emotion-language interactions above the level of explicit emotion words, and the role of peripheral feedback in comprehension. For this special issue exploring frontiers in the role of the body and environment in cognition, we propose a theory in which facial feedback provides a context-sensitive constraint on the simulation of actions described in language. Paralleling the role of emotions in real-world behavior, our account proposes that (1) facial expressions accompany sudden shifts in wellbeing as described in language; (2) facial expressions modulate emotional action systems during reading; and (3) emotional action systems prepare the reader for an effective simulation of the ensuing language content. To inform the theory and guide future research, we outline a framework based on internal models for motor control. To support the theory, we assemble evidence from diverse areas of research. Taking a functional view of emotion, we tie the theory to behavioral and neural evidence for a role of facial feedback in cognition. Our theoretical framework provides a detailed account that can guide future research on the role of emotional feedback in language processing, and on interactions of language and emotion. It also highlights the bodily periphery as relevant to theories of embodied cognition.

## Introduction

Language can cause powerful and reliable changes in the emotions of readers. A best-selling novel induces similar patterns of emotions across millions of independent readers. Yet, language is ambiguous at every level of analysis (Quine, 1960). How, in the face of this pervasive ambiguity, does language reliably influence our emotions? Proposed constraints in language understanding have ranged from innate, universal knowledge structures (Fodor, 1975, 1983) to probabilistic interaction between levels of linguistic representation (Kintsch, 1988).

For this special issue exploring frontiers in the role of the body and environment in cognition, we propose an alternative framework for describing interactions of language and emotion in which emotion constrains language processing through interactions between central systems for language and emotion processing, and the emotional periphery. In particular, we propose that facial feedback provides a context-sensitive constraint for guiding simulation of actions described in language. By the periphery, we mean aspects of the peripheral nervous system most closely associated with the emotions – the peripheral nerves and musculature of facial expression. The idea of peripheral constraints in high-level cognition is not new, although early peripheral theories of cognition made only limited progress (e.g., McGuigan, 1966).

Initial support for the account comes from embodied theories of cognition (Glenberg, 1997; Barsalou, 1999) that propose overlapping neural systems for processing both emotions and language about emotions (e.g., Niedenthal, 2007). The hypothesis that language about emotions will engage the same neural systems involved in real-world emotional experience is supported by research showing that lexical processing on words that directly name emotions (happy, sad, etc.) can be affected by emotional states (Niedenthal et al., 1997), and that strongly emotional words activate central circuitries of emotion (Citron, 2012). However, because existing theories have focused on language at the lexical level, they can’t readily explain effects of emotions in language that doesn’t explicitly describe emotions. While some parts of the neural systems for emotion and language may overlap, others may be dissociated, and natural discourse likely includes all possible combinations. Here we focus only the most difficult case for a theory of language and emotion – the case where genuine emotion is felt at the periphery even though the driving sentence does not contain an emotion word. This approach allows us to account for findings that are not easily explained by existing accounts of emotion and language, and it generates novel predictions about the interaction of emotion and language.

Our account differs from previous embodied theories by focusing on how emotion influences language processing above the lexical level. Rather than proposing a common neural substrate for emotion and language, we suggest that emotion states influence the simulation of actions described in language. We articulate this claim by building on mechanistic theories of motor control and simulation that explicitly provide a role for peripheral feedback in ongoing behavior. Doing so allows us to explain evidence that emotion states impact language that is not explicitly emotional. Previous accounts are unable to explain such evidence because they fail to consider how emotion impacts language above the lexical level, and because they rely on the claim about overlapping neural systems for emotional language and states of emotion.

The account carries three important assumptions about how emotion interacts with written language (although the account may also apply to verbal language understanding). All three assumptions are based on a functional view of emotion (e.g., Frijda, 1986, 2007; Levenson, 1994; Keltner and Gross, 1999; Barrett, 2006) that propose emotions produce physical changes in the body for guiding effective actions in the world. First, facial expressions accompany sudden shifts in wellbeing as described in text, much as they accompany sudden shifts in wellbeing in real-world situations. Second, facial expressions modulate emotional action systems during reading, much as they modulate emotional action systems in real-world behavior. And third, emotional action systems prepare the reader for an effective simulation of the ensuing language content, much as they prepare the organism for effective real-world actions. In short, peripheral expressions of emotion constrain language comprehension, just as they constrain effective actions.

To support the theory, we have organized the paper into two halves that each focus on one of its main claims. The first half addresses the claim that the emotional periphery has a functional role in language comprehension. We draw on research regarding the role of bodily feedback in language comprehension, evidence for emotion-language interactions from embodied cognition, and evidence from facial feedback theories of emotion. We give special attention to a recent theory of language, the Action-Based Language (ABL, Glenberg and Gallese, 2012) theory that provides a mechanistic framework for describing peripheral-central interactions in language processing. To elaborate the theory, we consider modifications of the ABL framework that lead to testable predictions for future study. The second half of the paper addresses the claim that emotions constrain language comprehension. We review evidence that emotion constrains action, cognition, and simulation, and we address the neural systems that are likely involved in this function. We begin by reviewing the evidence from embodied theories of language comprehension.

## A Role of the Periphery in Language

### Embodied theories of emotional language comprehension

Embodied theories of cognition provide a straightforward explanation for the close link between language and emotion. These theories suggest that language processing involves a mental simulation grounded in bodily and neural states of action, perception, and emotion (Glenberg, 1997; Barsalou, 1999; Havas et al., 2007). By simulation, such theories generally mean a representation of the situations, objects, or events described in text that is instantiated in the same neural systems used in original experience. By grounding, it is meant that semantic processing involves modality-specific symbols, rather than abstract, arbitrary, or amodal symbols as proposed by classical theories of language (Barsalou, 1999). Thus, language about action and perception involves the same neural and bodily systems used in action (Glenberg and Kaschak, 2002; Hauk et al., 2004) and perception (Pecher et al., 2004; Kaschak et al., 2005; Tettamanti et al., 2005; Rüschemeyer et al., 2010).

To develop the claim that comprehension of emotional language involves emotion simulation, Havas et al. (2007) measured the time needed to comprehend sentences describing emotionally laden events when the participant was in a matching or mismatching emotional state. Sentences, while emotional, made little or no reference to emotion states. An example pleasant sentence is, “You and your lover embrace after a long separation.” An unpleasant sentence is, “The police car pulls up behind you, siren blaring.” They covertly manipulated emotion using a procedure developed by Strack et al. (1988) which involves holding a pen in the mouth to produce either a smile (holding the pen using only the teeth) or a frown or pout (holding the pen using only the lips and not the teeth). This procedure has been shown to reliably influence positive and negative emotional experiences in the absence of conscious mediation (Adelman and Zajonc, 1989). They expected an interaction such that the processing of pleasant sentences would be faster when the pen is held in the teeth (and participants are smiling) than when the pen is held in the lips (so that smiling is prevented), and vice versa for the time to process unpleasant sentences. This is precisely what was found, both when participants were asked to judge the emotionality of the sentences, and when they were asked to simply read the sentences.

Why should being in a particular emotional state facilitate comprehension of the sentence? As suggested above, one possibility is that simulation occurs at the lexical level. Emotion words might activate central emotion systems that are potentiated by a matching emotional state (but not by a mismatching emotional state). This account is consistent with lexical priming theories of emotion-cognition interactions (Bower, 1981, 1991), in which the pen manipulation activates an emotion concept (e.g., “happy”), which then primes words associated with that emotion. Words that occur in pleasant sentences might elicit more positive emotional activation or less negative emotional activation than words that occur in unpleasant sentences.

In a subsequent experiment, Havas et al. (2007) used the pen manipulation in a lexical decision task to test the lexical priming account of their findings. They used words taken from their stimulus sentences that were rated as being “central to the meaning of the sentence,” as well as strongly emotional words taken from an emotion-word database. Although lexical decision times for words were speeded when preceded by semantically associated words (a classic priming effect), they were not speeded by the pen manipulation. Thus, a simple mood-priming account based on facial feedback is unlikely to explain the results.

Here, we develop an alternative, supra-lexical account of emotion simulation that focuses on the role of the peripheral-central interactions in grounding emotional language. We propose that emotion states of the body are called upon in real-time processing of emotional language, and that feedback from these states helps constrain subsequent simulation of the language content. Although we agree that modality-specific systems are involved in language processing, and that partially overlapping neural systems are involved in both emotional experience and emotional language processing, this account differs from previous accounts in two ways: first, it provides a framework for examining emotion-language interactions above the lexical level and second, it extends emotional grounding beyond central processing systems to account for influences of the emotional periphery.

Our account begins by integrating evidence for peripheral influences in language and emotion.

### Evidence from embodied theories of cognition

How strong is the evidence for a role of the periphery in language comprehension? There is evidence from motor cognition research that peripheral action systems play a part in simulation (e.g., de Lange et al., 2006), but the equivalency of simulation in motor imagery and language processing is unclear (Willems et al., 2009). While embodied theories of language have provided strong evidence for interactions in the central nervous system between linguistic and non-linguistic neural processes, evidence for peripheral influence in language processing is weaker. For example, Zwaan and Taylor (2006) asked participants to turn a dial clockwise or counterclockwise as they read through a text. When the required hand movement conflicted with the action described in the text (e.g., “turn the volume down low”), the phrase took longer to read. The authors explain this finding in terms of ideomotor theories (e.g., Greenwald, 1970) in which the idea of an action (reading the sentence) potentiates its execution. Presumably, peripheral activity interacts with simultaneous central motor planning processes involved in imagining the actions conveyed by the sentence, although explanations based on central motor planning processes are also plausible.

A stronger example is based on a study of the impact on perceptual judgments of lifting actions, which are heavily shaped by proprioceptive feedback (Hamilton et al., 2004). Observers lifted a weight while they simultaneously judged a weight being lifted in a video. When the observers’ weight was lighter than that in the video, they tended to overestimate the observed weight, and when their weight was heavier, they tended to underestimate the observed weight. This finding is surprising because it runs counter to the intuitive prediction that one’s own movements should prime the interpretations of another’s actions. Instead, the results demonstrate a repulsion effect where the neural feedback of an action is dedicated to one task (lifting a weight), it is presumably unavailable for another task (visual judgment of weight), and this biases the perceptual judgment in a direction away from the current action.

A similar repulsion effect in language comprehension was reported by Scorolli et al. (2009). They tested for an impact of sentence processing on lifting actions. A priming based account would predict that a sentence describing the lifting of a light object (e.g., pillow) would prime underestimates of the weight and result in faster lifting, whereas a sentence describing the lifting of a heavy object (e.g., tool chest) would prime overestimates of the weight, and slower lifting. After participants heard a sentence describing the lifting of a light object, they tended to lift light boxes slower (as if they overestimated the weight) and heavy boxes faster (as if underestimating the weight), and vice versa for sentences describing the lifting of a heavy object. While it’s possible that the interactions occur solely in central processing, these findings suggest that simulation in language comprehension is sensitive to concurrent feedback from the body.

More compelling evidence that peripheral feedback plays a functional role in language comprehension comes from two studies using emotional language (Havas et al., 2010). First, electromyographic recording of facial muscle activity (EMG) during language comprehension showed that comprehension of emotional language generates corresponding emotional facial expression. Muscle activity was recorded from the specific facial muscles for producing angry and sad expressions (corrugator supercilii), and happy facial expressions (orbicularis oculii, and zygomaticus majoris) while participants read angry, happy, and sad sentences, and pressed a button when the sentence had been understood. The dependent variable of interest was the activity of the three muscle groups between sentence onset and when participants pressed a button indicating they had read it. Stimulus sentences made little or no reference to emotions or emotion states: an example of a happy sentence is “The water park is refreshing on the hot summer day,” a sad example is “You slump in your chair when you realize that all of the schools rejected you,” and an angry example is “The pushy telemarketer won’t let you return to dinner.”

**Figure 1:**
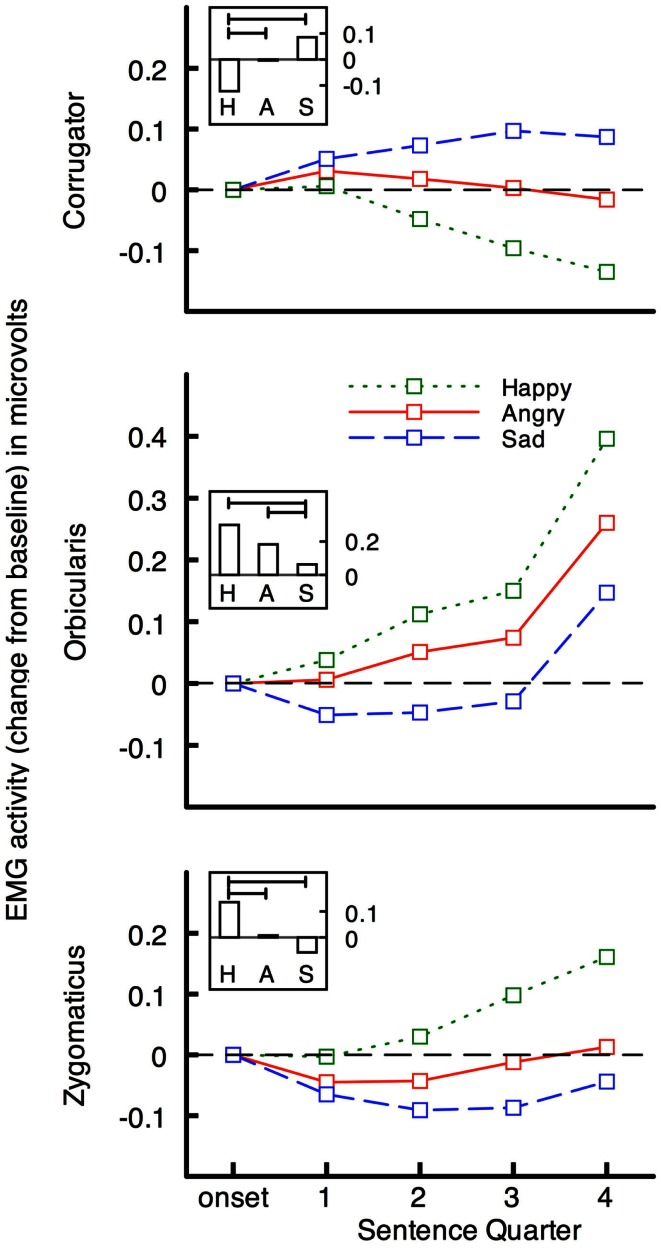
**Facial EMG change in microvolts from baseline (1000 ms before sentence onset) for emotional sentences across sentence quarters, and overall (inset; vertical bars represent mean EMG change during sentence presentation, and horizontal bars indicate significant comparisons) from Havas et al., 2010**. Activity in muscles for frowning (corrugator) and smiling (orbicularis and zygomaticus) diverges rapidly after onset of happy, angry, and sad, sentences. The fourth sentence quarter corresponds to participants' pressing of a button to indicate they understood the sentence. Sentence presentation durations have been standardized.

As predicted, facial muscles responded in an emotion congruent way to the sentences (see Figure 1). In the corrugator (frown) muscle, activity was greater for sad and angry, than for happy, sentences and vice versa in orbicularis and zygomaticus (smiling) muscles. Moreover, although the average reading times were several seconds, the muscular differentiation occurs rapidly – within 1000 ms of sentence onset.

A second, critical experiment asked whether peripheral feedback from emotion expression has a functional role in understanding emotional language. That is, does peripheral feedback from the facial expression contribute to language processing? For the study, first-time cosmetic surgery clinic patients about to receive botox injections in the corrugator muscle for treatment of glabellar (frown) lines were recruited. There were two reading sessions, just before botox injection and then 2 weeks after, wherein participants read the angry, sad, and happy sentences used in the above EMG experiment. Botox is a highly potent neurotoxin that causes temporary muscle denervation, and blocks muscle feedback by preventing release of acetylcholine (ACh) from presynaptic vesicles at the neuromuscular junction. Botox has also been shown to affect the intrafusal junction, reducing tonic afferent discharge (Rosales et al., 1996). Muscle relaxant effects result from the decrease in extrafusal muscle fiber activity and muscle strength within 1–3 days of injection, with peak weakening at around day 21 (Pestronk et al., 1976). It was predicted that paralysis of the muscle used in expressing emotions of anger and sadness would selectively affect comprehension of angry and sad, but not happy, sentences. As predicted, paralysis of the corrugator muscle selectively slowed comprehension of angry and sad sentences relative to pre-injection reading times, but happy sentences weren’t affected.

This finding provides strong evidence for peripheral emotional feedback in language comprehension, but it is consistent with two accounts of emotion simulation. First, botox could have influenced participants’ mood, perhaps by releasing them from anxiety, and this change in mood differentially primed the words found in the emotional sentences. This mood-congruency account is consistent with that of Bower (1981, 1991) and Niedenthal (2007) in that secondary, central changes in mood state drive the observed interaction. However, mood measures taken at each reading session showed no change in negative affect and a decrease in positive affect. Thus, the evidence supports a second account: that emotional feedback constrains simulation of the actions and events described in the language.

### Evidence from facial feedback theory

Support for this conclusion comes from facial feedback theories of emotion. Darwin (1872/1998) laid the foundation for research on the role of feedback in emotion, stating “The free expression by outward signs of an emotion intensifies it. On the other hand, the repression, as far as possible, of all outward signs, softens our emotion” (p. 22). William James (1884) directed attention of emotion researchers to the autonomic nervous system (ANS) and viscera as a source of emotions, initiating a vigorous debate about the informational adequacy of the viscera in producing differentiated emotional feelings. However, James (1884) had intended to include motor, as well as visceral, feedback in his theory (p. 192). Allport (1924) carried this idea forward, suggesting that autonomic patterns differentiated only pleasant and unpleasant emotions, but that the somatic system further distinguished emotions within each broad class.

Tomkins (1962) and Gellhorn (1964) were the first to emphasize a crucial role of facial feedback in emotion experience. Tomkins argued that because the nerves of the face are more finely differentiated, they provide more rapid and complex feedback to central brain mechanisms than do the viscera. He also noted that facial expressions precede visceral changes during an emotion episode. Gellhorn (1964) suggested a neurophysiological route via the hypothalamus by which finely tuned facial feedback influenced cortical processing of emotion. Izard (1977) further contextualized the role of facial feedback by describing it as a necessary, but insufficient, component of emotion experience. Still, he agreed that differentiation in consciousness of emotions depends on the rapid and specific sensory feedback from the face.

Paul Ekman (1992) updated James’ model of emotion, proposing that emotional situations trigger facial reactions, which then trigger specific patterns of autonomic response, and the combined somatic and autonomic patterns constitute emotional states. A good deal of evidence supports Ekman’s view. First, Robert Levenson and colleagues have provided strong evidence that distinct emotional facial expressions produce differential ANS activity (Ekman et al., 1983; Levenson et al., 1990; Levenson, 1992). They used the directed facial action task, in which participants are instructed to pose their face into a prototypical facial expression. As a result, the subjects show emotion-specific ANS patterns, and report experiencing the expressed emotion (Levenson et al., 1990). In addition, similar facial responses are observed across diverse cultures (Ekman and Friesen, 1971; Ekman, 1972), suggesting that facial expressions reflect a universal, functional adaptation.

This function may be inherently social. A proposal from social cognition research suggests that emotional expressions may transmit automatically across individuals through a mechanism of “emotional contagion” (Hatfield et al., 1994). Studies have shown that observing facial expressions automatically activates facial mimicry in the observer’s expressions (Dimberg, 1982; Hatfield et al., 1994), even in response to subliminally presented stimuli (Dimberg et al., 2000). Thus, feedback from the mimicry of another’s emotion expression may produce a similar emotion state in the observer, allowing for the automatic and implicit convergence of emotions across individuals. Neuroimaging studies show that areas consistently found to be involved in both observation and execution of facial expression include emotional processing regions of the brain, like the amygdala, insula, and cingulate gyrus, as well as motor areas (Molenberghs et al., 2012). Recent efforts to focus on the neural correlates of automatic facial mimicry (as opposed to mere observation) have combined brain imaging with facial EMG. So far, these studies have reliably found automatic facial mimicry to engage the same emotional brain networks, including the amygdalar region, insula, and cingulate cortex (Schilbach et al., 2008; Heller et al., 2011; Likowski et al., 2012). The relevance of these brain areas to the present theory will be discussed in greater detail below.

Despite the strong evidence for a causal role of facial expressions in emotional processing, theorists differ as to whether this relationship is due to facial feedback (Tomkins, 1962; Laird, 1974; Izard, 1991), or facial efference (motor output). For an instance of the latter, Ekman (1992) argues for a central, direct connection between motor cortex and other brain areas involved in coordinating physiological changes. The controversy has persisted mainly because these two possibilities have been very difficult to separate experimentally, although progress may be made through methods that manipulate facial feedback more precisely (i.e., with botox; Havas et al., 2010). For example, a neuroimaging study showed that botox-induced paralysis of the corrugator muscle 2 weeks prior to an facial expression imitation task reduced activation in neural centers involved in emotion processing (namely, amygdala, and orbitofrontal cortex), relative to activation in the same subjects before injection (Hennenlotter et al., 2009). In addition, they found that botox treatment reduced the functional connectivity of the amygdala with the dorsolateral pons, a brain stem region implicated in control of autonomic arousal (Critchley et al., 2001). Results of this type provide convincing evidence for the role for facial feedback in modulating central circuitries of emotion.

An important recent finding is that facial feedback effects may be largest during processing of ambiguous emotional stimuli. This idea echoes those of earlier theorists that assign facial feedback to tasks involving more finely differentiated emotions (e.g., Allport, 1924; Izard, 1977). Using a quasi-experimental design, Davis et al. (2010) compared self-reported emotions in subjects who chose facial botox injections to subjects who chose control injections that do not paralyze the facial muscles. Subjects rated their reactions to emotional video clips of varying valence and intensity both before and after injections, but because injections were administered in muscles used in both positive and negative emotions, results were interpreted only in terms of the overall magnitude of emotional experience rather than the valence. Overall, they found that botox injections reduced the magnitude of emotional response to the video clips relative to control injections (of cosmetic filler that doesn’t affect muscle activity). However, the reduction occurred only for video clips of mild positive intensity and not for strongly positive and negative clips. The authors suggest that the emotionality of strongly emotional clips is over-determined by responses of other, perhaps visceral, emotion systems.

Another recent study demonstrates that manipulation of facial feedback (both blocking and enhancing) impacts processing of ambiguous emotional stimuli – in this case, pictures of emotional faces. On the basis of findings that facial mimicry enhances perception of emotions in the faces of others (Goldman and Sripada, 2005), Neal and Chartrand (2011) asked participants to decode the expressions in pictures of faces. The facial expression stimuli were ambiguous in this task because they were completely obscured except for the region directly surrounding the eyes. In their first study, they compared the effects in patients with botox injections in facial muscles to those in a control injection that did not impact facial feedback. Accuracy in emotion perception was lower in patients whose facial feedback was blocked. In a second study, the authors determined that the reverse was also true by amplifying facial feedback using a restricting facial gel that produces muscle resistance, known to increase proprioceptive feedback. Performance accuracy in the emotion recognition task increased relative to control participants, but this difference was absent in controls tasks that are not supposed to involve facial mimicry.

In sum, the evidence from embodied cognition and from facial feedback theory suggests a functional role for facial feedback in emotion processing tasks, and perhaps particularly in tasks that involve automatic processing of emotionally ambiguous stimuli. Given this evidence, there are at least two ways in which facial feedback might influence a simulation of emotional sentences. First, facial feedback might contribute to a simulation by generating activity in modality-specific (i.e., emotional) systems of the brain. For example, feedback from a frown might potentiate the neural systems involved in sad or angry moods, and would thus enhance the recognition of language describing sadness or anger. However, this account fails to explain the absence of an effect of the pen in processing individual words from the study of Havas et al. (2007). Furthermore, this account fails to explain the absence of mood-congruent changes in the study of Havas et al. (2010). Evidence that botox injections selectively impact mood in non-clinical subjects is scant^1^. One study shows that patients who received botox injections in the frown muscle report normal levels of depression and anxiety compared to patients receiving other cosmetic surgery treatments who score in the borderline morbid range on these measures (Lewis and Bowler, 2009). However, because this study was correlational in design, participant self-selection cannot be ruled out. An alternative account that is consistent with the functional view of emotions outlined here is that emotion feedback allows context-sensitive modulation of perceptions, actions, and the simulation of actions in language. In this account, facial feedback contributes a highly sensitive source of information about the affective potential of the linguistic context that serves to constrain action simulation.

To understand how emotional feedback might constrain the simulation of action, we turn to a language-processing framework that explicitly provides a role for feedback in language: Glenberg and Gallese’s (2012) Action-based Language theory (ABL). The ABL theory is based on internal models framework of motor control in which bodily feedback contributes to the acquisition and updating of an internal representations for motor control. Glenberg and Gallese show how the framework (and peripheral feedback) can be applied to language, and they provide an explicit definition of simulation in language comprehension. After describing this work, we propose a modification of the ABL model for emotional language comprehension. By building on the ABL model, we aim to firmly ground our account in theories of action and to be explicit in our assumptions.

### The internal models framework

Computational approaches to motor control propose that the brain uses internal model for the control of behavior (Wolpert et al., 2001, 2003). Forward models (or, predictors) provide a model of the relation between a motor command and the sensory (vision, proprioception, touch, etc.) consequences of that movement. The function of a predictor is to predict these sensory consequences so that, given a particular motor command, the sensory outcome can be anticipated. A predictor might model, for example, the sensory consequences of lifting a cup to drink (that it will be heavy with water).

On the other hand, inverse models (or, controllers) do the inverse – they compute the context-sensitive motor commands necessary to accomplish a particular goal. A controller might model, for instance, the trajectory, and velocity of arm movements needed to lift a cup to drink. A biologically plausible account of how controllers are formed is through feedback error learning (Kawato, 1990, 1999). FEL uses performance error, or the difference between the desired and actual trajectory, for learning how to control movements. Much like the cruise control in a car, a controller monitors sensory feedback and continually adjusts motor output in order to maintain the desired outcome. Through this feedback error computation, the controller learns a functional mapping from motor commands to goal-based actions.

**Figure 2:**
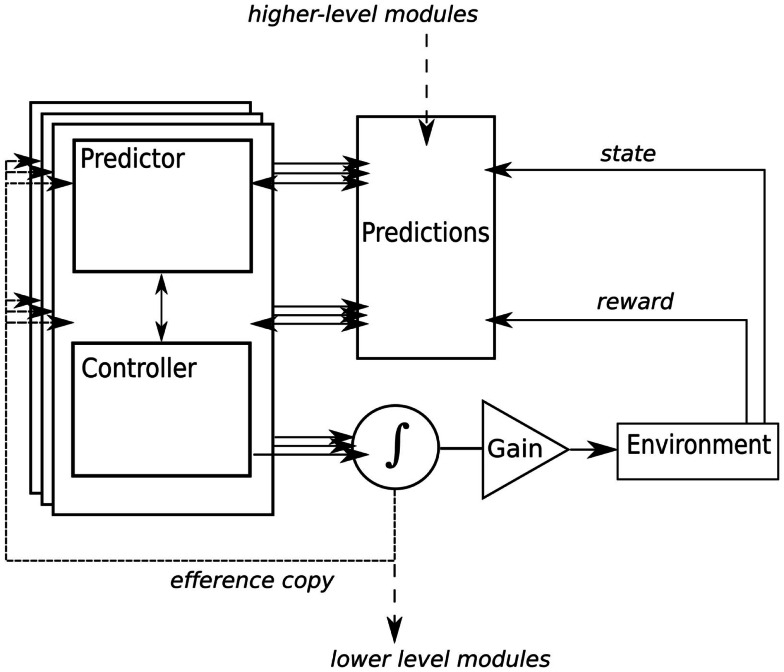
**A simplified internal models framework based on Glenberg and Gallese’s (2012) ABL model**. Here, we add a signal for learning to predict the reward of actions. Multiple modules, composed of paired predictors and controllers, anticipate the sensory and affective consequences of actions. Prediction error, derived from the actual sensory and affective consequences, drives learning in the controller and adjusts the responsibility for a particular module. As in Glenberg and Gallese's model, actual motor output is a weighted function of modules, higher-level modules provide hierarchical control of goal-based actions in the form of prior probabilities that influence lower-level module selection, and a gain controller is added for simulation in language comprehension.

For control of simple actions like reaching for a cup, multiple predictor-controller pairs, or modules, are used (see Figure 2; Wolpert and Kawato, 1998; Wolpert et al., 2003). But even simple actions are ambiguous. For example, lifting a cup when it is full has different dynamics than when the cup is empty, so different modules will be needed for each contingency and several modules may initially become activated^2^. The actual motor command is a weighted function of the outputs from the active controllers where the weighting of each controller is determined by two factors: the prior probabilities that each module is actually appropriate for the current context (the object affords action; Gibson, 1979), and the posterior probability, which is determined by prediction error. For example, if the selected module was not correct, then the prediction error will be large and this will decrease the module’s responsibility weighting. Thus, bodily feedback provides an ongoing signal for deriving contextually appropriate actions in real-time motor control. Bodily feedback be particularly important when dealing with novel contexts. Recent evidence suggests that feedback gains are increased during early stages of learning when the appropriate controller is ambiguous (Franklin et al., 2012).

For goal-based actions, Wolpert and colleagues (Haruno et al., 2001) have proposed that higher-level modules for goal-based action (say, drinking) learn to coordinate a sequence of lower-level actions, like reaching to grasp, lifting a cup, and taking a drink. The higher-level controller generates prior probabilities that lower-level modules are needed, while the higher-level predictor predicts the posterior probabilities that lower-level modules are accurate. This hierarchical organization reflects the neural architecture of the motor control system where at higher cortical levels the motor system is organized into actions rather than individual movements (e.g., Umilta et al., 2008). This feature allows the motor system to create combinations of elementary units that are contextually appropriate, or that satisfy multiple simultaneous constraints. It has been noted that both language and motor control share this quality (McClelland et al., 1988).

### The action-based language theory

In their ABL theory, Glenberg and Gallese (2012) propose that the same solution used in motor control was exploited through evolution by language. They link language and action through the neural overlap between the mirror neuron system for action and Broca’s area in the inferior frontal cortex (IFG) for speech articulation (see also Fadiga et al., 2006). The mirror neuron system encodes motor intentions (either observed or executed), including the motor intentions behind heard or observed speech acts. Because in human development, motor actions often co-occur with speech (e.g., a parent might say the word for an action while they demonstrate that action to a child) speech articulation primes motor action, and vice versa, through associative Hebbian learning. For example, the module for articulating a word like “drink” is associated through social development with the module for the motor actions involved in drinking. Likewise, language about a “girl” activates the module used to predict the sensory consequence of moving the eyes to see a girl illustrated in a children’s book.

For their model of language comprehension, Glenberg and Gallese add a gain control for the gating of sensory feedback, and for inhibiting motor output in “offline” simulation, imagery, planning, practice, and language (see Figure 2). For example, if the gain is set to inhibit motor output, but the predictor is free to make sensory predictions, then the output resembles mental imagery^3^.

As Glenberg and Gallese illustrate, the ABL model gives an account of simulation in comprehending a sentence like, “The girl takes the cup from the boy.” First, motor output gain is set low to avoid literally acting out the actions described in the language. Upon hearing words for objects or individuals (e.g., “The girl”), speech action controllers are activated, which in turn activate the associated action controllers for interacting with those objects or individuals. Output from the controller produces a prediction of the sensory consequences of such interaction, akin to mental imagery of the objects or individuals. Upon hearing verbs (e.g., “takes”), speech controllers pass activation to multiple possible action controllers that could fulfill the goal of the action (e.g., reach to grab, and the controller is selected according to the prior probability that it can fulfill the goal. After processing an image of “the cup,” selection of the next controller is weighted by prior probabilities for the objects of such actions (e.g., “from the boy” affords receiving a cup, whereas “from the tree” does not). Importantly, the prior probability assigned to each action controller depends in part on the action’s affordances (Gibson, 1979) for fulfilling the higher-level goal conveyed by the language (to drink). As Glenberg and Gallese put it, “comprehension is the process of fitting together actions suggested by the linguistic symbols so that those actions accomplish a higher-level goal …” (Glenberg and Gallese, 2012, p. 12–13). If goal-based actions can’t readily be integrated (“The girl takes the cup from the tree”), then comprehension is challenged.

### Internal models for emotional language

Although not addressed by Glenberg and Gallese, we believe the internal models account of comprehension carries an important additional implication regarding cases where comprehension is challenged, or in the language of the framework, where there is a failure to select modules that fulfill the higher-level goal of the language. Such cases should result in performance error and a consequent adjustment in controller output. In online motor control, feedback from such controller output provides contextual information for adjusting the unfolding action. In online language understanding, controller output could serve a similar function for guiding an unfolding simulation. Context should be particularly useful when the actions needed to simulate the meaning of the sentence are ambiguous, or underspecified in the language. Context, which we take here to mean the current state of body-world interactions (or affordances), helps to guide the selection of an appropriate controller. Thus, the model suggests that language will call on the body when comprehension is challenged by underspecified affordances for action-object integration.

This implication suggests a way that emotions interact in language. The following proposal rests on the assumption that emotions accompany a sudden change in wellbeing relative to the current state, and that they automatically lead to actions that can capitalize upon, or mitigate, that change (see also Frijda, 1986, 2007). To illustrate this assumption, imagine encountering a bear while walking in the woods. The experience would automatically engage modality-specific neural systems, including emotion systems that motivate actions. Quickly, both the body and brain would be reconfigured for taking adaptive actions. And because the body has changed, the affordances of the situation have changed: a walking stick in your hand may now be readily perceived as a potential weapon for defense. As this scenario illustrates, the most effective action in an emotional situation is determined by the combination of changes in bodily preparation for action, and the affordances provided in the environment. The neurophysiological bases for such changes are discussed in the following sections.

In understanding a sentence, affordances for effective action must be provided by the language. We propose that language that describes a change in the state of wellbeing that invites but under-specifies effective action will make module-selection difficult, and this will lead to an increase in motor output in the form of facial patterns that reflect an estimate of the affective change described in the language (e.g., improvement or decline is reflected by a smile or a frown, respectively)^4^. For example, a reader can only infer the most effective actions when understanding the meaning of a sentence like “The water park is refreshing on the hot summer day.” Effective actions might include wading, and splashing in the water – actions that would allow someone to capitalize on the potential for relief from heat, as implied by the sentence. Because understanding the language requires that the reader infer these actions (they are not made explicit in the language), the result will be facial afference in the form of a smile. By extension, language that describes a shift in the state of wellbeing in which effective action is over-determined may not elicit facial efference. The effective actions in the sentence, “You slam on the brake and curse when a driver cuts you off,” are already well specified. Although the language is emotional in both cases, we hypothesize that the former sentence should lead to greater facial efference than the latter.

Although our account is speculative, the previous sections have reviewed a wide range of evidence for a key feature of the theory – a role for the emotional periphery in language comprehension. The following sections review a wide range of support for a second key feature of the theory – that emotion constrains language comprehension. To bolster the claim, we first show how emotion constrains action, cognition, and simulation. We then address the neural bases for emotion constraints in language comprehension before we consider additional features of the theory.

## Emotion Constrains Language Comprehension

### Emotion constrains action

Most likely, emotions evolved to prepare organisms for effective actions. When we are angry, our fists clench, our heart rate is increased, and we are prepared for aggressive or defensive actions. When sad, our posture deflates, our heart rate decreases, and we experience loss of energy. In short, our emotions constrain our future possibilities for action.

Early emotion theorists recognized that different emotions correlate with distinct changes in the body. Following James (1884) infamous emotional feedback theory in which he equated bodily feedback with the subjective experience of emotion, appraisal theorists (e.g., Arnold, 1960; Frijda, 1986, 2007) proposed implicit cognitive processes that mediate an emotional stimulus and bodily response. On the other hand, strong theoretical arguments (Zajonc, 1980; Murphy and Zajonc, 1993) and neuroscientific evidence suggest that emotional situations can organize action systems directly without any intervening cognitive processing.

Working in rats, LeDoux (1996, 2002) identified the amygdala as a critical structure in mediating fear learning. The central nucleus of the amygdala initiates fear responses, including freezing, escape, and autonomic changes, and the basal nucleus projects to motor circuits in the ventral striatum where information about an aversive stimulus contributes to action selection (Alexander and Crutcher, 1990). Because the pathway from thalamus to the amygdala bypasses the cortex and is thus more direct than the cortical route, it provides a neural mechanism by which emotional situations directly influence emotional behaviors, bypassing cognitive processes.

Regardless of whether amygdala activation from emotional stimuli arises in humans via direct or indirect pathways (for debate on this question, see Pessoa and Adolphs, 2010; Cauchoix and Crouzet, 2013), the critical finding for the present purpose is that activity in the amygdala appears to correspond to changes in the current state of wellbeing. In monkeys, the amygdala has been shown to be highly sensitive to the value of a reward relative to the current state of the body (Paton et al., 2006; Belova et al., 2007, 2008). In humans, a similar mechanism has been demonstrated with a procedure called backwards-masking, where an emotionally arousing stimulus is presented very briefly and is then followed by a neutral stimulus that blocks the emotional stimulus from entering consciousness. Such unconsciously presented fearful stimuli have been shown to cause increases in skin conductance and heart rate that reflect autonomic arousal (Esteves et al., 1994). The specific brain changes that occur during unconscious emotion processing have been examined by combining the backwards-masking procedure with fMRI. When participants in an fMRI scanner are presented with pictures of either fearful or happy faces for a subliminal duration, followed by neutral faces, the subliminally perceived emotional faces cause differential activity in the amygdala (Whalen et al., 1998). Fearful masked faces increased amygdala activity, whereas the happy faces decreased amygdala activity. Thus, cross-species evidence indicates that emotional stimuli organize action system immediately, sometimes unconsciously, to fulfill the goals at hand. Action is central in emotion in part because emotional responses are implemented in the form of action tendencies, or bodily responses that potentiate adaptive actions. That is, emotions constrain bodily actions.

There is evidence that the amygdala also responds to changes in wellbeing that are signaled by symbolic or linguistic stimuli. Phelps et al. (2001) told participants that they might receive an electric shock to the wrist paired with one stimulus (a blue square), but that another stimulus (a yellow square) signaled that no shock would occur. Using fMRI, they found that presentations of the symbol connoting threat preceded activation of the left amygdala, which correlated with the physiological expression of fear learning. They also found a correlation between the expression of fear and activity in the left insula, an area involved in cortically representing the affective state of the body. This suggests that the left amygdala is involved in the expression of fears and associated bodily states that are imagined through the use of symbols. Amygdala activation has consistently been observed in response to the presentation of emotional words (reviewed in Citron, 2012), and during reading of emotionally intense narratives (Wallentin et al., 2011).

Based on this association with emotional language comprehension in humans, we propose that the amygdala encodes changes in wellbeing described in language. For example, amygdala responses to reading about a sudden improvement in outlook (“Incredibly, the numbers drawn all match those on the ticket in your hand”) marshal autonomic (perhaps parasympathetic) resources involved in joy, whereas amygdala responses to reading about a sudden decline in wellbeing (“Your grandmother had a stroke and is in critical condition”) elicits other, perhaps sympathetic, changes in the ANS). These autonomic modulations serve to constrain the possibilities for action, and thus constrain the possibilities for action simulation.

A defining feature of emotions is that their effects are often systematic, phasically influencing a range of actions in a hierarchical manner (Panksepp, 1998). The ANS regulates cardiovascular, gastrointestinal, electrodermal, respiratory, endocrine, and exocrine organs in support of action responses to challenge and opportunity (Levenson, 1992, 2003). Several theorists have proposed that emotions are organized at higher functional levels, constituting two basic motivational circuits (Lang et al., 1990; Davidson, 1992; Gray, 1994). For example, Lang and Bradley have proposed that emotions are organized around two motivational systems, appetitive and defensive, mediated by distinct systems at cortical and limbic levels (Lang et al., 1990; Bradley et al., 2001). In terms of actions, this division translates roughly into behaviors of approach and withdrawal, respectively, where appetitive activation generally leads to approach behaviors, and defensive activation generally leads to withdrawal behaviors (Davidson, 1992, 1995, 1998).

An important consequence to this hierarchical organization is that emotions constrain actions in a probabilistic, rather than deterministic, manner. Top-down emotional constraints on action will be modified by bottom-up constraints of the environment. Thus, emotion states don’t correspond to specific actions, but rather something much like action tendencies, so that the same emotion state may lead to categorically related but unique actions depending on the particular context. For instance, at the highest level of organization, motivational engagement of the defensive system may prompt different emotion states like fear or anger, depending on whether the situation calls for flight or fight (Lang et al., 1990). And at a lower-level of emotional action, anger may or may not lead to striking out, depending on whether the confrontation escalates or is averted. Thus, effective actions are jointly influenced by underlying emotion states and the sensorimotor affordances that arise in the situation (Gibson, 1979). In our formulation, these joint functions are served by the global autonomic changes elicited by the face, and the simulation of action as guided by the language. Next, we discuss evidence and theory that emotion is capable of constraining cognition.

### Emotion constrains cognition

Several theorists have proposed that emotion systems help guide cognitive processes (Pribram, 1969; Nauta, 1971; Damasio, 1994). Here we only briefly discuss one kind of cognition: decision-making. Damasio and colleagues observed that patients with lesions in the prefrontal cortex (ventromedial prefrontal cortex, VMPFC) were severely impaired in personal and social decision-making, and in particular have difficulty in anticipating future positive and negative consequences of their actions, in spite of otherwise preserved intellectual abilities, including language (Damasio, 1979, 1994). Their decision-making is often slow and error prone, and sometimes random and impulsive. However, immediately available rewards and punishments do influence their behavior. Whereas most people show increased skin conductance (a measure of autonomic arousal) in anticipation of a risky choice, even before they explicitly know the choice is risky, VMPFC patients do not.

To account for the pattern of deficits, Damasio et al. (1991), Damasio (1994) proposed a somatic marker hypothesis in which the components of a complex experience are recorded in modality-specific neural systems, and these records become associated with the emotional response that occurred during the experience. The VMPFC is responsible for learning the associations between a complex situation (e.g., walking in the woods and encountering a bear) with the accompanying emotion state (e.g., fear), and for reactivating the emotion state when components of the original experience are later encountered (e.g., seeing the walking stick by the door might reactivate feelings of fear). This function is valuable in that it provides an implicit emotional “marker” which signals the value of each decision before action is taken. Emotion reactivation can occur via a “body-loop,” whereupon the viscera actually change and the ensuing changes are relayed to somatosensory cortices, including the insula. Or, emotional changes can occur via an “as-if-body-loop” where signals are conveyed directly to the cortex, bypassing the physiological changes. Together, the insula and anterior cingulate gyrus may be important in integrating cortically mediated cognitive functions with somatosensory and autonomic changes (see also Medford and Critchley, 2010).

When do decisions engage the “body-loop” or “as-if-body-loop”? Bechara and Damasio (2005) suggest the “body-loop” becomes increasingly important under circumstances of uncertainty or ambiguity. For example, normal subjects generate little skin conductance responses during tasks that involve decision-making under relative certainty, compared to tasks involving decision-making under ambiguity. It is intriguing to note the parallel with the internal models framework in which peripheral feedback is particularly important during learning of tasks with novel (ambiguous) dynamics.

By providing a representation of “what it feels like” to be in a particular situation, a somatosensory pattern in the insula may be particularly important in constraining a simulation of actions. First, through strong projections to the amygdala, the insula can modulate actions by influencing ANS changes. Second, the emotional somatosensory pattern helps to constrain the process of reasoning over multiple options and future outcomes by marking the sensory components, which describe a related scenario of future outcome, as good or bad. Somatic states influence cognitive processing by acting as a biasing signal, and can be used to rapidly accept or reject certain option-outcome pairs. Without this function, the decision process would depend entirely on logic operations over many option-outcome pairs, which is slower and may fail to account for previous experience – just the pattern of behavior seen in VMPFC damaged patients.

Damasio (1994) proposes that emotional representations for use in social communication have their own distinct structure, the anterior cingulate cortex (ACC), stemming from observations of patients with damage in this area. Whereas damage to the face area of the motor cortex will impact the ability to voluntarily make a smile, it spares the ability to make a genuine, spontaneous smile. Conversely, emotion-related movements originate in the ACC, and patients with damage to this area show abnormal spontaneous facial expressions of emotion, but normal voluntary facial movement.

Damasio’s proposed mechanism by which somatic state representations influence cognition is through the activation of neuromodulator nuclei that project to cortical networks. Bechara and Damasio (2005) hypothesize that the biasing action of somatic states on response selection is mediated by the release of the major neurotransmitter systems, dopamine (DA), serotonin (5-HT), noradrenalin (NA), and ACh whose nuclei are located in the brainstem. Changes in neurotransmitter release induced by somatic state signals modulate the synaptic activities of cortical neurons subserving behavior and cognition, thereby providing a mechanism for somatic states to exert a biasing effect on cognition. In their account, these two neural systems of emotion (neuromodulation and somatic markers) interact to provide predictions about “what it feels like” to engage in particular actions. Ascending neuromodulators facilitate computation of future rewards given the current state of the body, thereby constraining action selection in frontal cortices.

Although the somatic marker hypothesis has provided evidence for a constraining role of emotion in one kind of cognitive task that involves simulation (of future rewards in decision-making), additional evidence comes from tasks that more closely resemble language comprehension.

### Emotion constrains simulation

In the view we are presenting, emotional language calls upon emotion systems of the body that constrain a simulation of actions and events described by the text. Our view differs from other simulation accounts in that emotion simulation occurs even in the absence of explicit affective information like emotional words. That is, we assume readers will use their own emotional knowledge to make inferences based on described actions or events that are not explicitly emotional. Thus, readers bring to bear two sources of information in understanding language: external information provided by actions in the language, and internal information provided by an emotional inference mechanism.

This feature of our theory bears a resemblance to theories from several other areas of research, which we briefly mention here. First, discourse comprehension research shows that readers readily bring their knowledge of emotions to make inferences about story characters’ emotions (Gernsbacher and Robertson, 1992; Gernsbacher et al., 1992, 1998; Haenggi et al., 1993). Moreover, readers make such emotional inferences just as readily in the absence of explicit emotional information, simply from descriptions of story characters’ actions, as they are when emotional information is present (deVega et al., 1996; Gygax et al., 2007). Thus, our theory is congruent with research from discourse comprehension.

An important claim of our view is that readers’ emotions serve to constrain interpretation of the language. This idea can be traced back at least to “reader’s response” literary theorists who argued that the reader’s personal experiences provide the basis for textual understanding (Iser, 1978). Some empirical support for this notion is provided by theorists of literary appreciation (Miall, 1988, 1995; Miall and Kuiken, 1994) who argued that emotions play a primary role in appreciating literary stories. In one study (Miall, 1988), participants read short stories phrase-by-phrase while reading times were collected. Afterward, participants rated each phrase for its emotional significance (“Is feeling significant to this phrase?”), and correlations between reading times and affective ratings were measured. There was a positive correlation in the early part of the stories where readers are presumably using affect to guide a search after meaning. Correlations became negative later in the story, presumably because affect is now confirming the interpretations set up in the early part. Citing Damasio’s patients with VMPFC damage who are unable to select among possible response options, Miall (1995) speculates that in reading literature, this deficit might present as a failure to decide among possible inferences about a sentence in a story. However, because the methods used by literary theorists often focus on post-comprehension processes, they can’t speak to how emotional states are generated to begin with. As described above, our view is that facial expressions are generated at points of ambiguity.

Our theory also bears a resemblance to social cognition research into “mentalizing,” or the ability to explain and predict behavior of others in terms of one’s own mental or emotional states (Frith et al., 1991) and empathy, or the ability to share the feelings of others (Decety and Lamm, 2006). Because the mental states of others are not directly observable, they must be inferred solely on the basis of overt behaviors, or abstract (i.e., verbal) descriptions of those behaviors. Whereas emotional decision-making is associated with the VMPFC, mentalizing from verbal material (i.e., inferring the likely goals, intentions, and desires of people described in stories) reliably engages more dorsal regions of the medial prefrontal cortex (MPFC and the ACC), as reported in a large meta-analysis of neuroimaging studies (Van Overwalle, 2009). Just as somatic state representations in the insular cortex are well suited, both functionally and anatomically, to contribute to decision-making, they may serve to constrain the processes that take place in the MPFC (Augustine, 1996). Functionally, anticipated somatosensory states would provide an experiential basis for predicting the future behavior of others, in much the same way as they help guide one’s own subsequent behaviors.

Other research has shown that somatic state representations in the insula might provide a basis for empathy. Neuroimaging studies have shown that the same regions of the insula are active both during experience of aversive events, such as disgust (Wicker et al., 2003) and pain (Singer et al., 2004), and during the observation of those states in others. Overlapping activity in the insula across these divergent modes of experience is thought to indicate a neural mechanism for emotional understanding, and provides initial support for somatic state representations in inferring others’ emotions (Wicker et al., 2003).

### Neural bases of emotion constraints in language

In previous sections, we have mentioned the neural circuits involved in some aspects of our theory. Here, we address two remaining questions. First, how are facial responses elicited by neural processing of sentences? While this question is unexplored in the neuroscientific literature, we propose that facial responses arise in response to sentences that convey a sudden change in wellbeing relative to the current state of the body, and under-specify the appropriate course of action, driving emotional action inferences. Such sentences may produce a state of cognitive conflict about which actions are appropriate for fulfilling the goals in the language. Take the sad sentence (written by an undergraduate research assistant for our EMG and botox studies), “You slump in your chair when you realize all the schools rejected you.” For the present purpose, we can consider the higher-level goal of the sentence to be a simulation of the dejection, anguish, and exasperation (and the correlated actions) associated with social and vocational disappointment. Simulating the initial action of the sentence (slumping) will generate a modality-specific prediction of the sensorimotor consequences of the action, including a prediction of withdrawal, or perhaps pain (MacDonald and Leary, 2005), in somatic cortices. But because the reader’s actual current somatic state (alertness and engagement as required by the reading task) conflicts with the somatic prediction, a large prediction error will result, forcing a shift in action controllers to simulate the higher-level goal of the sentence. However, effective actions are not specified in the remainder of the sentence, and so the ensuing simulation is faced with a conflict. Here, we propose that a facial expression will be triggered that reflects the direction of the somatic prediction error (a frown). The resulting context-sensitive facial feedback will modulate the emotional state of the body (as described above), and update the somatic state representation for use in simulation^5^.

We consider the cingulate cortex a likely substrate for mediating facial efference because it is strongly associated with task performance under cognitive conflict (Botvinick et al., 2004), is proposed to underlie the integration of cognitive and emotional processes (Bush et al., 2000), and contains direct projections to the facial nucleus (as recently demonstrated in monkeys; Morecraft et al., 2004). Tasks that involve cognitive conflict elicit facial activity (Schacht et al., 2009). And while positively and negatively valenced words elicit subgenual cingulate cortex activity (Maddock et al., 2003), repetition of emotional words produces a clear habituation response (as reported in Maddock et al., 2003), suggesting that novelty of the emotional stimulus might be important. Interestingly non-verbal emotional stimuli (pictures of facial expressions) do not activate subgenual cingulate cortex (e.g., Maddock, 1999), perhaps because they convey affective meaning directly, whereas emotional words involve a higher degree of semantic inference.

Next, how might somatic state representations constrain action simulation during language comprehension? Given the strong bidirectional connection between the anterior insula and inferior frontal gyrus (IFG), which includes Broca’s region in humans (Mesulam and Mufson, 1985; Augustine, 1996), we see at least two possibilities. One is that they provide a modality-specific neural substrate for the representation of emotion states described in language, as predicted by other emotion simulation accounts (Havas et al., 2007; Niedenthal, 2007). If so, then the same region of the insula should be active during both language about emotion and during real emotion. Accordingly, Jabbi et al. (2008) found that a region of anterior insula (extending to inferior frontal operculum) became active when the same participants either felt disgust, saw facial expressions of disgust, or read short passages describing a disgusting situation. The functional overlap supports simulation theories of social cognition in general, although interesting differences between the three conditions were observed in the connectivity findings. Reading passages about disgust uniquely included Broca’s area in the left IFG.

A second possibility is that somatic state representations encode autonomic constraints of the body that differentially affect the simulation (and execution) of some actions over others, much as autonomic constraints influence real actions. Thus, somatic state representations would help resolve ambiguity in action simulation. If so, then we would expect that current body states can become rapidly incorporated into online language comprehension processes. Indeed, behavioral evidence has shown that bodily constraints on action are incorporated within early stages of syntactic ambiguity resolution (within 500 ms) during sentence comprehension (Chambers et al., 2004). The insula has a long-standing role in language-related motor control (Dronkers, 1996). A neurodegenerative disease that impacts both the insula and language is progressive non-fluent aphasia (PNFA). Patients with (PNFA) are selectively impaired in sentence comprehension, but spared in single-word comprehension, and other non-linguistic cognitive abilities (Peelle et al., 2008). Although a role of insular cortex in resolving ambiguity during sentence comprehension has yet to be explored systematically, extant data support such a role.

## Unique Features of the Theory

Although our account is speculative, it differs from other accounts of emotion simulation in language and thus makes unique predictions. Foremost, emotion influences language processing above the lexical level. Rather than provide a common neural substrate for emotion and language about emotion (e.g., Niedenthal, 2007), somatic state representations influence a simulation of actions as driven by speech action controllers in Broca’s area. This account remains congruent with embodied theories of language comprehension because the outputs of action controllers are predictions in modality-specific regions of the brain (Barsalou et al., 2003; Pulvermuller, 2005), and because emotion state constrain a modality-specific simulation. Although the two accounts of emotion simulation make differing predictions, we don’t believe they are mutually exclusive, and are rather likely to operate in tandem during online language comprehension.

The model offers an explanation for a range of empirical observations on the interaction of emotion and language comprehension. For example, in the study of Havas et al. (2007), we found an interaction of emotion and language comprehension: body states of emotion (smiling, and frowning) that are congruent with the emotional meaning implied in the sentence facilitate comprehension, whereas emotion states that are incongruent hinder it. Consider reading one of the Angry sentences from that study, “After the fight with the stubborn bigot, you slam the car door.” The negative emotional expression produced by holding the pen in the lips activates associated negative state representations (angry or sad) in somatosensory cortices, biasing the selection of effective actions (e.g., aggressive, or defensive actions). Because the body is prepared to produce the kind of actions that are required for understanding the sentence, a simulation of the second half of the sentence (“you slam the car door”) is completed with ease. By contrast, a positive somatosensory representation produced by holding the pen in the teeth would hinder the simulation of such actions.

This account also explains emotional interactions during language comprehension when there is no pen to force a facial expression. Here, simulating the action in the sentence produces somatic prediction error, and generates an emotional response in preparation for subsequent understanding. For example, the initial phrase in the sentence, “You slump in your chair when you realize that all of the schools rejected you” will generate emotional afference compatible with the initial decrease in wellbeing. This is the result we found using EMG (reported in Havas et al., 2010).

Finally, we can explain how blocking facial afference that is congruent with the emotionality of a sentence might hinder comprehension. Despite any facial afference generated in processing the angry and sad sentences, botox prevents negative facial feedback from modulating central emotion circuits that would otherwise constrain the simulation. But because happy expressions are unaffected, they are free to modulate central circuits of emotion, and constrain the simulation of happy sentences.

By way of comparison with Glenberg and Gallese’s (2012) ABL model, we too assume that the solution used in motor control for deriving emotionally appropriate action was exploited through evolution by language. For language, our theory works much like the ABL model in that modules are grounded in actions and sensory predictions, a gain control mechanism suppresses literal execution, and controller output is tantamount to a simulation. However, there are also several features that are new in our theory. Foremost, selection of modules for running a simulation of language is determined not just by motor prediction error, but also by a somatic error signal. Thus, an extension of the ABL model for emotional language comprehension would add a forward model that learns to predict future somatic states that result from actions. Action controllers for simulation are jointly determined by the operation of both types of predictor that work in a complementary way to determine the relative goodness of particular actions. Where the predictors are uncertain, the reward model can guide behavior, and vice versa. When effective actions are underspecified in the language, emotion simulation will guide the derivation of those actions. This feature may have implications for comprehension of abstract concepts, and may explain why concepts that bear on a person’s wellbeing but that don’t specify particular action, like “freedom” or “justice,” are often emotionally evocative.

Another difference between the ABL theory and ours is that lower-level control structures are constrained by higher-level emotion states. That is, global states of emotion (that correspond to action tendencies of approach or withdrawal, for example) will constrain the simulation of actions in a probabilistic fashion. Because emotional facial expressions change action tendencies through modulation of the ANS (Levenson, 1992), they predispose the body for taking certain actions. For example, a positive emotion state will potentiate actions of approach (Davidson, 1992). If language understanding requires a simulation of similar such actions, then comprehension will have been facilitated. Thus, because smiling will potentiate actions of approach and affiliation, it is likely to facilitate a simulation of the actions in a sentence like, “You lean over your birthday cake and blow out all the candles.”

Finally, our account gives emotion a central role in language comprehension, even for simulation of language that is only implicitly emotional. We think this is fitting – language conveys emotional meaning at every level of analysis, from prosody, to morphology, to syntax (Majid, 2012), and a reader’s emotions are engaged by language at the earliest stages of processing (Van Berkum et al., 2009).

## Limitations and Future Directions

Our purpose in this article is to provide a theoretical synthesis of research from several domains, with an emphasis on recent, and intriguing findings. By necessity, we have overlooked vast areas of work that deserve consideration, and only mentioned some work that deserves deeper consideration. Further refinement of the theory will depend on a more careful accounting of this work. For example, our theory bears a similarity to accounts of facial expression recognition in which facial feedback provides a source of automatic, rapid, and unconscious constraints on processing (e.g., Dimberg et al., 2000). Another important, and fast-developing body of research that deserves greater attention surrounds the notion of “simulation.” Our focus on the mechanisms of emotion simulation may have overlooked broader developments in this area are likely to bear on the present theory.

Another limitation concerns our treatment of alternative accounts for facial feedback effects. One important alternative rests on changes in mood, and studies have demonstrated that facial feedback can influence mood, and mood processes (e.g., Kleinke et al., 1998; Duclos and Laird, 2001). While we have developed our theory partly in an effort to account for evidence against this hypothesis (see Havas et al., 2010), mood-based explanations will need to be carefully considered in future empirical validation of the theory.

Much of our theory derives from the internal models framework, and its recent projection to language in the ABL model of Glenberg and Gallese (2012). Although in its present form the account is an advance in that it suggests how simulation in Broca’s region may modulated by emotion systems, much work is needed to establish the validity of the ABL theory, and to connect it with emotional language comprehension. Although many details are still to be worked out, we consider this a step toward specifying interactions of emotion and language that have long interested researchers, and whose existing empirical connection is currently only tenuous.

We have claimed that our theory supports simulation-based accounts of language comprehension (Glenberg and Kaschak, 2002; Barsalou et al., 2003; Pulvermuller, 2005) by providing a mechanism by which emotion influences action controllers in LIFG for driving the simulation of modality-specific actions and perceptions (as described by Glenberg and Gallese, 2012). Our account is embodied in that understanding language involves a simulation of meaning in multimodal brain areas that correspond to the referents in the language. Language results from the operation of controllers (which learn to derive actions from sensory goals) and predictors (which learn to predict the sensory consequences of those actions) in LIFG. Thus, understanding the meaning of the word “clap” involves first deriving the speech module (in Broca’s area) for uttering the word “clap” from the text, and then generating sensory predictions of the actions (in pre-motor and motor cortex) and the sounds (in auditory cortex) involved in clapping. As generated by facial feedback, emotion states (in the insula) constrain the selection of controllers and predictors to facilitate simulation of the language content. Thus, simulation is grounded in action, perception, and emotion.

Although LIFG is not always implicated in simulation theories of language (but see Pulvermuller, 2005), we believe this region is important for embodied theories for two reasons. First, LIFG is critical in syntax, and any theory that fails to account for this involvement is necessarily incomplete. Second, an important challenge for embodied theories is to explain predication, or conceptual combination into grammatically meaningful statements. We believe that the present theory contributes to the grounding of predication in action and emotion.

For future work, one promising feature of the model is that it suggests a constraint on the creativity of the human conceptual system. Recall that somatic prediction error signals the relative value of taking a particular action in a particular context, and can be used for action selection. Specifically, the signal corresponds to the predicted change in emotional state resulting from the action, as represented in somatic cortices. This signal is likely to be important in guiding the combination of concepts during language comprehension. Glenberg and Robertson (2000) suggested that conceptual combination is constrained by the affordances of the objects described in noun-verb combinations. They presented participants with sentences describing novel situations that ended in one of three ways, and participants judged the sentences as sensible or nonsense. For example, the phrase “Bill needed to paint the top of the barn wall so he stood on his …” could be followed either by “ladder,” “tractor,” or “hammer.” They found that sentences ending with objects that afforded accomplishing the goal but that were used in an unusual way (tractor) were judged as sensible just as readily as sentences ending with objects that both afforded the goal and were used in a typical way (ladder). Yet, sentences ending with non-afforded and unusual objects (hammer) were quickly judged as nonsense, despite the fact that the word “hammer” was similar to the word “tractor” in many other ways (both are strongly associated with the context, both are tools, both are common words, etc.). Thus, they argued that conceptual combinations are constrained by whether the affordances of objects in the language can be meshed in service of reaching goals.

A benefit from our approach is that it helps to differentiate conceptual combinations that may equally afford goal-obtainment, but differ in the emotional value with which they do so. For example, standing on a tractor may not be as expedient or safe as standing on a ladder to paint the top of a barn. By contrast, the somatic error signal helps to differentiate these options on the basis of their value for the organism. Actions that afford success more expediently (i.e., they deliver the reward of goal attainment more directly, with greater certainty, or more quickly) will be understood more readily, subject to the current emotional state of the reader. Thus, the present model enriches Glenberg and Robertson’s (2000) account without reverting to standard, amodal linguistic criteria commonly used to explain semantic combination effects (word frequency, animacy, typicality, etc.).

## Conclusion

By selectively blocking muscle feedback, botulinum toxin-a (botox) has allowed researchers a new opportunity to test the role of the body in cognition. Recent experiments with emotional facial feedback have shown that botox modulates emotion experience and its neural centers, and selectively affects emotion-language comprehension, thereby strongly supporting facial feedback theories of emotion and embodied accounts of cognition.

Using a functional account of emotion, we explored implications of this research for a mechanistic understanding of the body’s role in language, and proposed a role of bodily feedback in providing context-sensitive constraints on language processing. Paralleling the role of emotions in real-world behavior, our account proposes that (1) facial expressions accompany sudden shifts in wellbeing as described in language; (2) facial expressions modulate emotion action systems during reading; and (3) emotional action states prepare the reader for an effective simulation of the ensuing language content. In language comprehension, modules in Broca’s area learn to predict the emotional consequences of simulated actions, and prediction error leads to facial afference. Facial feedback provides context-sensitive modulation of visceral states, and these emotional state changes become represented in somatosensory cortex. In turn, somatic representations constrain simulation of actions and action inferences for deriving the meaning of the language. By selectively blocking emotional feedback, botox systematically affects the simulation value of actions and perceptions described in the language. Our theoretical framework, based on internal models, provides a detailed account that can guide future research on the role of emotional feedback in language processing, and on interactions of language and emotion. It also highlights the bodily periphery as relevant to theories of embodied cognition.

## Conflict of Interest Statement

The authors declare that the research was conducted in the absence of any commercial or financial relationships that could be construed as a potential conflict of interest.

## Footnotes

^1^Although evidence for mood changes from conscious, self-generated expressions is stronger (e.g., Duclos and Laird, 2001), these data may not bear on the specific, unconscious mechanisms we are able to isolate with botox. Furthermore, any mood changes due to facial feedback may be secondary to the amygdala-mediated changes that we propose occur in language processing.

^2^Although modules are unnecessary in dynamical systems approaches to motor control (e.g., Churchland et al., 2012), Wolpert and Kawato (1998) discuss several advantages to using a modular approach. One important advantage for present consideration is that language is modular, or conveyed by discrete units in the form of phonemes or words. Productivity in language is accomplished by combining these discrete units, from different levels, in novel ways (Hockett, 1960), much as Wolpert and Kawato propose for the production of novel movements.

^3^In most cases, the gain control inhibits most movement, but some movement may not be completely inhibited, as seen in gesture that accompanies speech. Gesture research has shown that tasks that involve more strongly simulated actions are more likely to evoke speech-accompanying gestures (Hostetter and Alibali, 2008, 2010), even when communicative demands are held constant across tasks.

^4^Because facial muscles produce tonic afferent discharge, a decrease in facial muscle output would be informative for module-selection as well.

^5^If the predicted somatic state error is small, then a shift in the action controller may not be necessary. For example, if the reader is already in a somatic state congruent with the language, then comprehension processes are predicted to proceed with facility in the absence of facial afference.
